# Disturbed bone marrow adiposity in patients with Cushing’s syndrome and glucocorticoid- and postmenopausal- induced osteoporosis

**DOI:** 10.3389/fendo.2023.1232574

**Published:** 2023-10-10

**Authors:** Nina N. Sørensen, Christina M. Andreasen, Pia R. Jensen, Ellen M. Hauge, Jens Bollerslev, Jean-Marie Delaissé, Moustapha Kassem, Abbas Jafari, Marta Diaz-delCastillo, Thomas L. Andersen

**Affiliations:** ^1^ Research Unit of Pathology, Department of Clinical Research, University of Southern Denmark, Odense, Denmark; ^2^ Department of Pathology, Odense University Hospital, Odense, Denmark; ^3^ Department of Molecular Medicine, University of Southern Denmark, Odense, Denmark; ^4^ Danish Spatial Imaging Consortium (DanSIC), Denmark; ^5^ Clinical Cell Biology (KCB), Vejle/Lillebaelt Hospital, Institute of Regional Health Research (IRS), University of Southern Denmark, Vejle, Denmark; ^6^ Department of Rheumatology, Aarhus University Hospital, Aarhus, Denmark; ^7^ Department of Clinical Medicine, Aarhus University, Aarhus, Denmark; ^8^ Section of Specialized Endocrinology, Oslo University Hospital, Oslo, Norway; ^9^ Faculty of Medicine, University of Oslo, Oslo, Norway; ^10^ Department of Cellular and Molecular Medicine, Novo Nordisk Foundation Center for Stem Cell Biology (DanStem), University of Copenhagen, Copenhagen, Denmark; ^11^ Molecular Endocrinology & Stem Cell Research Unit (KMEB), Department of Endocrinology and Metabolism, Odense University Hospital & University of Southern Denmark, Odense, Denmark; ^12^ Department of Forensic Medicine, Aarhus University, Aarhus, Denmark

**Keywords:** bone, bone marrow adipocyte (BMAd), bone marrow adipose tissue (BMAT), Cushing´s syndrome, glucocorticoids, osteoporosis, post-menopausal osteoporosis

## Abstract

**Background:**

Skeletal stem/progenitor cells (SSPCs) in the bone marrow can differentiate into osteoblasts or adipocytes in response to microenvironmental signalling input, including hormonal signalling. Glucocorticoids (GC) are corticosteroid hormones that promote adipogenic differentiation and are endogenously increased in patients with Cushing´s syndrome (CS). Here, we investigate bone marrow adiposity changes in response to endogenous or exogenous GC increases. For that, we characterize bone biopsies from patients with CS and post-menopausal women with glucocorticoid-induced osteoporosis (GC-O), compared to age-matched controls, including postmenopausal osteoporotic patients (PM-O).

**Methods:**

Transiliac crest bone biopsies from CS patients and healthy controls, and from postmenopausal women with GC-O and matched controls were analysed; an additional cohort included biopsies from women with PM-O. Plastic-embedded biopsies were sectioned for histomorphometric characterization and quantification of adipocytes. The fraction of adipocyte area per tissue (Ad.Ar/T.Ar) and marrow area (Ad.Ar/Ma.Ar), mean adipocyte profile area (Ad.Pf.Ar) and adipocyte profile density (N.Ad.Pf/Ma.Ar) were determined and correlated to steroid levels. Furthermore, the spatial distribution of adipocytes in relation to trabecular bone was characterized and correlations between bone marrow adiposity and bone remodeling parameters investigated.

**Results:**

Biopsies from patients with CS and GC-O presented increased Ad.Ar/Ma.Ar, along with adipocyte hypertrophy and hyperplasia. In patients with CS, both Ad.Ar/Ma.Ar and Ad.Pf.Ar significantly correlated with serum cortisol levels. Spatial distribution analyses revealed that, in CS, the increase in Ad.Ar/Ma.Ar near to trabecular bone (<100 µm) was mediated by both adipocyte hypertrophy and hyperplasia, while N.Ad.Pf/Ma.Ar further into the marrow (>100 µm) remained unchanged. In contrast, patients with GC-O only presented increased Ad.Ar/Ma.Ar and mean Ad.Pf.Ar>100 µm from trabecular bone surface, highlighting the differential effect of increased endogenous steroid accumulation. Finally, the Ad.Ar/Ma.Ar and Ad.Ar/T.Ar correlated with the canopy coverage above remodeling events.

**Conclusion:**

Increased cortisol production in patients with CS induces increased bone marrow adiposity, primarily mediated by adipocyte hypertrophy. This adiposity is particularly evident near trabecular bone surfaces, where hyperplasia also occurs. The differential pattern of adiposity in patients with CS and GC-O highlights that bone marrow adipocytes and their progenitors may respond differently in these two GC-mediated bone diseases.

## 1 Introduction

The bone marrow adipose tissue (BMAT) fraction constitutes up to 70% of the adult bone marrow volume, along with trabecular bone and hematopoietic cells (1–3). While BMAT was long considered an inert bone marrow component, its crucial role in energy storage, endocrine function and bone metabolism has become increasingly acknowledged over the last few decades (1, 2). The cellular component of BMAT are adipocytes that differentiate from skeletal stem/progenitor cells (SSPCs), also known as bone marrow stromal cells (BMSCs), which can alternatively give rise to osteogenic-lineage cells (1, 4). Whether these multipotent SSPCs lean towards adipogenesis or osteoblastogenesis depends on complex signaling cascades initiated by microenvironmental stimuli. Because of this reciprocal relationship between adipogenic or osteogenic cell fate (5–7), it is not unexpected that conditions associated with decreased bone volume also present increased BMAT (8), such as aging (9, 10), obesity (11), osteoporosis (3, 12), and anorexia nervosa (13, 14).

In physiological conditions, the microscopic basic multicellular units (BMUs), which consist of a tightly regulated sequence of events including an initial osteoclastic bone resorption, a reversal-resorption phase and an osteoblastic bone formation phase, maintain bone homeostasis (15). On trabecular and endocortical bone surfaces, the BMUs operate on the bone surfaces separated from the bone marrow cavity by bone marrow envelope cells, forming a bone remodeling compartment canopy above remodeling sites (16–18). Several studies have proposed that this envelope/canopy is a local reservoir of osteoprogenitors that are delivered to eroded surfaces, where they can further differentiate into mature bone-forming osteoblasts (19). This model is further supported by numerous observations. In conditions such as multiple myeloma, endogenous CS, as well as PM-O and GC-O, an insufficient canopy coverage of the BMUs correlate with the accumulation of eroded surfaces. This delayed transition to bone formation is likely attributed to insufficient recruitment of osteoprogenitors to the eroded surfaces (20, 21). Nonetheless, the causes of this insufficient envelope/canopy coverage remain unknown. Since SSPCs play a crucial role in maintaining the influx of osteoprogenitors that compose the canopy, increased adipogenic differentiation might be associated with canopy disruptions. These disruptions, in turn, contribute to the observed delayed onset of bone formation seen in conditions such as osteoporosis and CS. Thus, SSPCs may pose an interesting target for the treatment of bone disease in osteoporotic conditions, such as in patients with CS.

CS is a rare disorder in which patients present abnormally high levels of endogenous cortisol. CS has an annual incidence of 1.8–3.2 cases/million population (22) and presents a varied etiology (e.g. pituitary tumor, adrenal tumor, adrenal hyperplasia) (23). Bone disease is a common morbidity in this disorder, as 50% of CS patients develop osteoporosis, and 30–50% experience fractures, particularly at the vertebral level (24, 25). In agreement with the adipogenic effect of GCs on SSPC differentiation (26–29), bones from CS patients present increased BMAT (25, 30) and we have previously reported canopy alterations that correlate with decreased bone formation surfaces in CS patients (20). However, the mechanisms behind BMAT accumulation and its effect on trabecular bone remodeling remain unknown.

Here, we investigate the effect of endogenous cortisol increases on BMAT and its effect on bone formation in patients with CS. Furthermore, we will compare it with the effect of exogenous glucocorticoids through a cohort of patients with GC-O.

## 2 Materials and methods

### 2.1 Patients and bone biopsy specimens

The study was approved by the Danish National Committee on Biomedical Research Ethics (S-20070121) and performed in agreement with the Declaration of Helsinki. All biological tissue was collected by skilled medical professionals based on informed consent.

A total of 62 bone biopsies were included in the study. All tissue originates from our two previous studies on premature loss of canopies and its association to deficient bone formation in patients with CS (20) and on GC-O (15, 21); immunostained slides have been reanalyzed in the presented manuscript. The following patient cohorts were included in this study; details of sex and age can be found in **Table 1**.

- Control (C): Middle aged healthy volunteers; n = 9 (20, 31–33).
- Cushing´s syndrome (CS): Middle aged patients diagnosed with CS and presenting increased endogenous cortisol serum levels of 400–1000 nmol/l; n = 18 (20).
- Elderly control (eC): Elderly postmenopausal healthy volunteers; n = 8 (15, 20, 21).
- Postmenopausal osteoporosis (PM-O): Elderly PM-O patients, who had experienced ≥1 low energy fracture and had not received any osteoporosis treatment; n = 14 (15, 21).
- Glucocorticoid-induced osteoporosis (GC-O): Elderly postmenopausal patients diagnosed with GC-O. Patients had received GC therapy for 0.6-15 years (mean duration of therapy: 6.6 years), with daily doses of 5–60 mg (average prescribed daily dose: 15.9 mg), including prednisone (n = 12) or prednisolone (n = 1) treatment. The underlying disorders requiring GC treatment included polymyalgia (n = 2), collagenosis (n = 1), temporal arthritis (n = 1), arthritis (n = 3), dermatitis (n = 1) and asthma (n = 5). All patients had experienced at least one low energy trauma leading to vertebral fracture, and have not received any osteoporosis treatment; n = 13 (20).

**Table 1:** Demographic patient information.

	Control (C)	Cushing syndrome (CS)	Elderly control (eC)	Post-menopausal osteoporosis (PM–O)	Glucocorticoid-induced osteoporosis (GC-O)
Women (n)	7	13	8	14	13
Age (median **±** SD)	54.00 ± 13.88	45.50 ± 11.20	69.00 ± 8.49	72.00 ± 5.64	73.00 ± 5.09
Men (n)	2	5			
Age (median **±** SD)	38.00 ± 12.72	38.50 ± 13.74			

### 2.2 Tissue processing

Ethanol-fixed, 7-mm trephine iliac crest bone biopsies were embedded in methyl methacrylate (MMA) and cut into 7-µm-thick sections for histomorphometric analyses. One section per biopsy was Masson-Goldner trichrome stained; briefly, deplastification was performed through slide immersion in 2-methoxyethyl-acetat (Merck, Denmark) for 45 min, followed by 5 min wash in 99% ethanol, 2 min wash in 96% ethanol and immersion in tap water. Slides were then stained with Weigert´s iron hematoxylin (Merck, Denmark) for 1 h, washed with tap water for 10 minutes, immersed in Ponceau/Fuchsin solution (Sigma and Merck, Denmark) for 15 min and washed in 1% acetic acid twice. Slides were then stained with fast green (Sigma, Denmark) for 20 minutes and washed twice with 1% acetic acid, before dehydration in 96% ethanol and 99% ethanol. Subsequently, slides were mounted in Pertex mounting media (Sigma, Denmark) and scanned at 20x on a NanoZoomer S360 scanner (Hamamatsu, Japan).

### 2.3 Histomorphometric analyses

**Figure 1:**
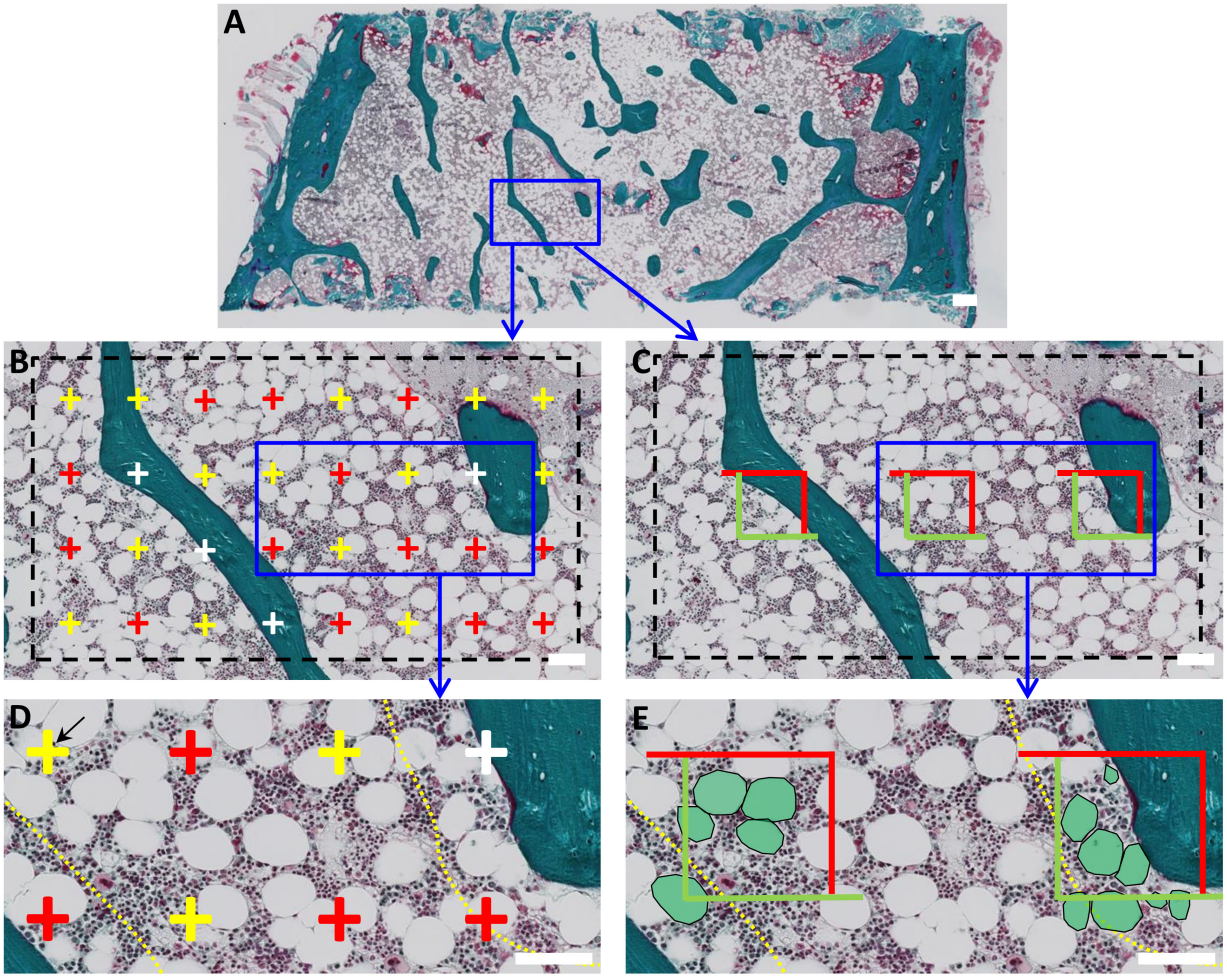
Histomorphometric characterization of BMAT in human bone marrow biopsies. **(A)** Digitalized images of Goldner Masson trichrome-stained bone biopsies were analyzed using two parallel approaches. **(B)** First, a point-grid was superimposed and the points that overlapped adipocytes (red), marrow (yellow) or trabecular bone (white) were quantified. Adipose area per tissue area (Ad.Ar/T.Ar) or marrow area (Ad.Ar/Ma.Ar) were calculated as the sum of points overlapping adipocytes divided by the sum of points overlapping the total tissue area or marrow area. **(D)** Grid-points were further subdivided according to their spatial distribution in relation to bone surface, i.e., within or above 100 µm from trabecular and endocortical bone. This boundary is indicated with a yellow dashed line. **(C)** In a parallel analysis, a box-grid containing three measuring boxes was superimposed to the slide to calculate Ad.Pf.Ar and N.Ad.Pf/Ma.Ar. **(E)** Adipocytes within the boxes boundaries and/or overlapping the green sides were included in the measurement. Again these BMAT parameters were divided relation to trabecular bone, i.e., within or above 100 µm of the bone surface. Boundary is indicated as a yellow dotted line. Scale bars: A is 1mm; B-E are 100 µm.

Tissue slides were pseudo-anonymized to a random code and analyzed using NDP.view2 viewing software (Hamamatsu, Japan), as illustrated in **Figure 1**. All measurements were performed by the same investigator, who was blinded in regard to study group and patient identity. The histomorphometric parameters assessed follow the standardized nomenclature recommended by the International Bone Marrow Adiposity Society (BMAS) and ASBMR (34, 35). The adipose area per tissue area (Ad.Ae/T.Ar) and marrow area (Ad.Ar/Ma.Ar) were estimated using a point-grid superimposed upon the digitalized tissue at 5x (**Figure 1B**). Briefly, the first visual field was randomly placed over one corner of the marrow cavity, and upon characterization of that area, it was subsequently moved along to systematically cover the entire marrow compartment. The Ad.Ar/T.Ar (%) was then estimated by dividing the sum of points that overlapped with adipocytes by the total sum of points that fell within the bone marrow cavity (**Figure 1D**). In contrast, the Ad.Ar/Ma.Ar (%) was estimated by dividing the sum of points that overlapped with adipocytes by the total sum of points that fell within the bone marrow cavity, omitting the trabecular bone compartment (**Figure 1D**). Next, the grid points were further divided according to whether they were covering? <100 µm or >100 µm of the bone surface (**Figure 1D**), to address whether the spatial distribution of adipocytes is affected by trabecular bone. A mean of 539 ± 247 grid-points were investigated in each section, amounting to a total of 33,419 grid-points among all biopsies.

The mean adipocyte profile area (Ad.Pf.Ar) and the number of adipocyte profiles per marrow area (N.Ad.Pf/Ma.Ar) were estimated using a box-grid superimposed upon the digitalized slide at 5x (**Figure 1D**). As described, the first visual field was randomly placed over a corner of the marrow cavity and moved along to systematically cover the entire tissue. In this case, the box-grid included 3 boxed areas delimited by two green and two red sides. The profile area of adipocytes located within or partly within the boxed areas, but not intercepted by the red exclusion sides, was measured (**Figure 1E**). Mean Ad.Pf.Ar (mm^2^) was estimated as the mean profile area of the investigated adipocyte profiles in each section. N.Ad.Pf/Ma.Ar (#/mm^2^) was calculated by dividing the number adipocyte profiles situated within the box-grid by the total area of the investigated boxes within the marrow cavity. As previously described, Ad.Pf.Ar and N.Ad.Pf/Ma.Ar were re-calculated according to whether they were <100 µm or >100µm of the bone surface (**Figure 1E**), to assess whether proximity to trabecular bone affects the adiposity characteristics.

The bone remodeling parameters were reused from a previous study on the same bone biopsies from CS and controls (20). In short, these bone remodeling parameters included the percentage of eroded surfaces (ES/BS) and osteoid surfaces (OS/BS) per bone surface, the percentage of canopy covered ES, OS and BMU (ES+OS). The parameters were morphologically estimated within the same blinded Masson-Goldner trichrome stained sections used for the analysis of the BMAT by a single observer validated by a senior observer.

### 2.4 Statistical analysis

Statistical analyses and plots were performed using GraphPad Prism v5 (GraphPad Software, Inc., La Jolla, CA, USA). Data were tested for normality using the D’Agostino-Pearson omnibus normality test. Differences between control and CS biopsies were determined using Student´s *t-*test, for parametric data, of Mann-Whitney test, for non-parametric data. Differences between elderly controls, PM-O and GC-O were calculated using one-way ANOVA followed by Bonferroni´s correction for multiple comparisons for parametric data, or the Kruskal-Wallis test followed by Dunn´s correction for multiple comparisons for non-parametric data. Spatial distribution of BMAT content was analyzed by two-way repeated measures ANOVA followed by Bonferroni correction. Correlations were analyzed using the Pearson or Spearman correlation test, as appropriate. All data are presented as individual and mean values ± standard error of the mean (SEM), and significance was set at p<0.05.

## 3 Results

### 3.1 Increased endogenous and exogenous glucocorticoid levels influence on BMAT content

Previous reports have shown that GCs promote the adipogenic differentiation of SSPCs, leading to increased BMAT fraction (26–30). To confirm these results, we characterized the BMAT content in trephine iliac crest bone biopsies of patients with increased endogenous cortisol (CS) and exogenous glucocorticoids (GC-O), compared with their respective controls.

**Figure 2:**
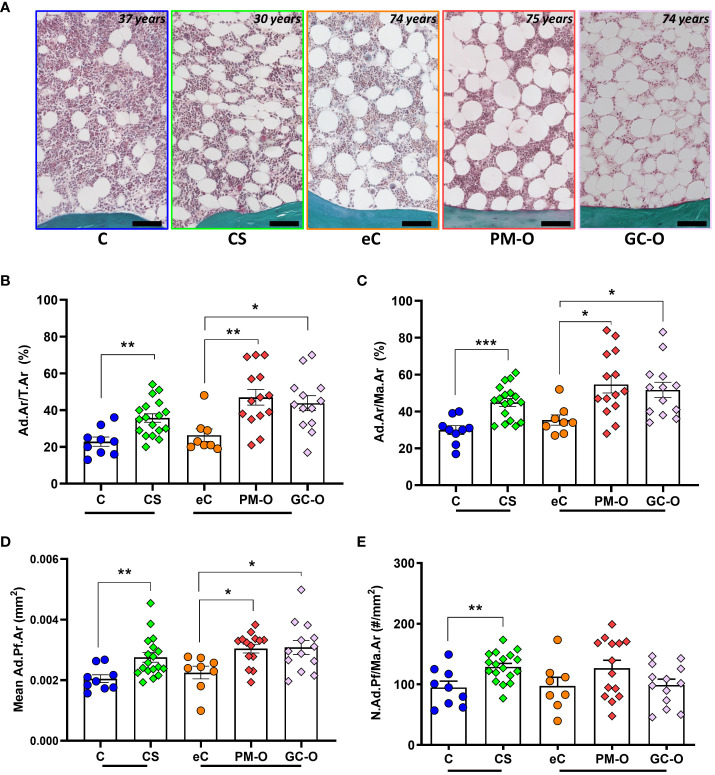
Effect of glucocorticoids on BMAT content. **(A)** Representative images of the bone marrow and its adiposity in CS patients and its control, as well as in PM-O and GC-O patients and their elderly controls. **(B–D)** CS patient, PM-O and GC-O have a significant higher Ad.Ar/T.Ar **(B)**, Ad.Ar/Ma.Ar **(C)** and mean Ad.Pf.Ar **(D)** compared to their respective controls. **(E)** Only CS patients, not PM-O and GC-O, have a significant higher N.Ad.Pf/Ma.Ar compared to their respective controls. All data are displayed as individual biopsies and mean ± SEM. C vs. CS data were analyzed by Student´s t-test or Mann Whitney test. eC vs. PM-O and GC-O data were analyzed by one-way ANOVA with Bonferroni correction or Kruskal-Wallis with Dunn´s correction. *p < 0.05; **p < 0.01; ***p < 0.001. Ad.Ar/T.Ar, adipose area per tissue area; Ad.Ar/Ma.Ar, adipose area per marrow area; mean Ad.Pf.Ar, mean adipocyte profile area; N.Ad.Pf/Ma.Ar, Number of adipocyte profiles per marrow area. C, Control; CS, Cushing´s Syndrome; eC, elderly control; PM-O, post-menopausal osteoporosis; GC-O, glucocorticoid-induced osteoporosis.

A mean of 84 ± 53 adipocytes were counted and measured in each section, amounting in a total of 5,230 adipocytes in all biopsies (**Figure 2A**). Both the Ad.Ar/T.Ar and Ad.Ar/Ma.Ar were significantly increased in CS and GC-O patients compared with their respective controls (**Figures 2B, C**) [Ad.Ar/T.Ar in C vs. CS: p = 0.0019 and Ad.Ar/Ma.Ar in C vs. CS: p = 0.0002 by two-tailed, unpaired t-test; Ad.Ar/T.Ar in eC vs GC-O vs PM-O: p = 0.027 by Kruskal Wallis, followed by *post-hoc* Dunn´s test, Ad.Ar/Ma.Ar in eC vs GC-O vs PM-O: p = 0.041 by one-way ANOVA followed by Bonferroni´s multiple comparison correction]. Similarly, adiposity was increased in patients with PM-O [Ad.Ar/T.Ar in eC vs PM-O: p = 0.0073 by *post-hoc* Dunn´s test and Ad.Ar/Ma.Ar in eC vs PM-O: p = 0.013 by Bonferroni´s correction] (**Figures 2B, C**). The mean Ad.Pf.Ar was significantly larger in CS and GC-O patients, as well as in PM-O patients (**Figure 2D**) [C vs CS: two-tailed Mann-Whitney, p = 0.0035; eC vs. GC-O vs- PM-O: p=0.022 by Kruskal Wallis, followed by Dunn´s multiple comparisons eC vs- GC-O p=0.041, eC vs. PM-O p=0.017], suggesting that hypertrophy is a common occurrence in increased glucocorticoid environments. However, adipocyte hyperplasia was only observed in patients with accumulation of endogenous (CS), but not exogenous (GC-O), GCs (**Figure 2E**) [C vs. CS: p = 0.0044 by two-tailed, unpaired t-test; eC vs GC-O vs PM-O: p = 0.16 by one-way ANOVA]. Overall, these results suggest that GC accumulation increases BMAT, but the mechanisms may differ depending on the hormonal source.

**Figure 3:**
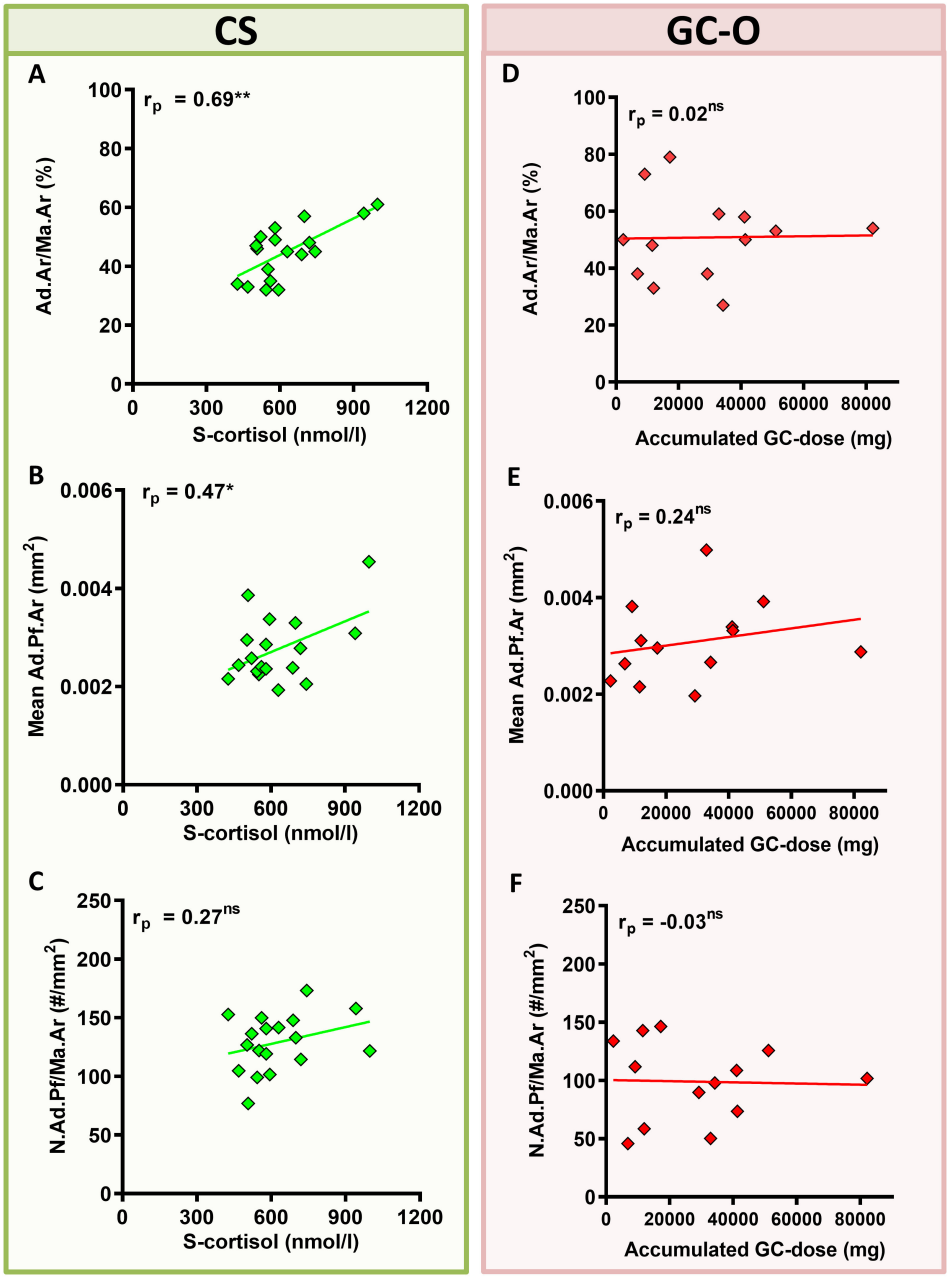
Correlation between glucocorticoid levels/accumulated dose and BMAT content. **(A–C)** Patients with CS displayed a significant correlation between serum glucocorticoid levels (s-cortisol) and Ad.Ar/Ma.Ar **(A)** and mean Ad.Pf.Ar **(B)**, but not N.Ad.Pf/Ma.Ar **(C)**. **(D–F)** Patients with GC-O showed no correlation between the accumulated glucocorticoid dose and the BMAT parameters. Symbols represent individual patients and solid lines indicate simple linear regression curves. Data are analyzed by Pearson (r_p_) correlation. *p < 0.05; **p < 0.01. ns, non significant.

Next, we investigated whether BMAT content was directly influenced by GC levels (CS) or accumulated GC-doses (GC-O) at the time of biopsy. The median accumulated GC dose in the GC-O group was 17.18 mg (2.25–186.60 mg) over a variable treatment period of 1.5 to 20 years (median treatment period: 6.25 years). In patients with CS, Ad.Ar/Ma.Ar was significantly correlated with serum cortisol levels (**Figures 3A, B**) (p = 0.0016), while the mean Ad.Pf.Ar and N.Ad.Pf/Ma.Ar were not (**Figure 3C**) [S-cortisol correlation to Ad.Pf.Ar and N.Ad.Pf/Ma.Ar p = 0.44 and p = 0.27, respectively]. In contrast, no correlation between accumulated glucocorticoid dose and Ad.Ar/Ma.Ar (**Figure 3D**) [p = 0.95], mean Ad.Pf.Ar (**Figure 3E)** [p = 0.43] or N.Ad.Pf/Ma.Ar (**Figure 3F**) [p = 0. 91] was found in patients with GC-O, which suggest potentially different effects of endogenous and exogenous glucocorticoids on bone adiposity.

### 3.2 Age influence on BMAT content

Because aging is a known risk factor for BMAT accumulation (9, 10, 36, 37), we investigated the correlation between age and adipose content, size and density in patients with increased GC levels. Our previous analyses included two different control groups stratified according to age (C vs. eC), due to the different median age of our main research groups (median age of CS is 45 ± 11 years, while median age of GC-O is 73 ± 5 years; see **Table 1**). To better evaluate the effect of aging on BMAT content, our analyses included a “mixed control (MixC)” group that encompassed both C and eC individuals.

**Figure 4:**
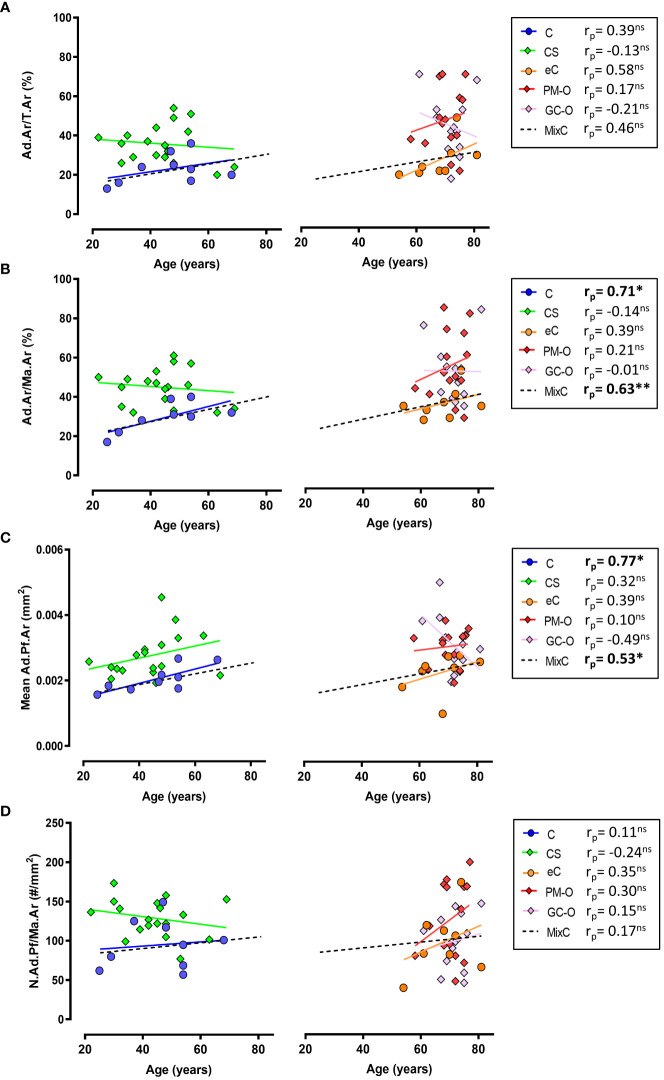
Correlation between aging and BMAT parameters. **(A)** Ad.Ar/T.Ar is independent of age in all the investigated groups except elderly controls (eC) and the mix control group, while **(B)** Ad.Ar/Ma.Ar is significantly correlated with age in C and MixC groups. **(C)** Adipocyte profile size is significantly correlated with age in C and MixC groups, while **(D)** N.Ad.Pf/Ma.Ar and aging show no significant correlation. Symbols represent individual patients and solid lines indicate simple linear regression analyses. Data are analyzed by Pearson (p_r_) r correlation. *p < 0.05; **p < 0.01. ns, non significant. Correlation slope and intercept *p*-values are listed in **Supplementary Table 1**.

Correlation analyses demonstrated that Ad.Ar/T.Ar and N.Ad.Pf/Ma.Ar are independent of age in all the included cohorts, except for an age-dependent increase in Ad.Ar/T.Ar in the eC group (**Figures 4A, D**). However, both Ad.Ar/Ma.Ar and mean Ad.Pf.Ar were significantly correlated with aging in the C and mixC groups (**Figures 4B, C**). A summary of slope and intercept p-values for these correlation analyses can be found in **Supplementary Table 1**. Overall, our data indicate that aging promotes adiposity, but is not a main cofounding factor for the differences in BMAT content observed in the CS, GC-O or PM-O cohorts.

### 3.3 Differential spatial distribution of BMAT in relation to trabecular bone

**Figure 5:**
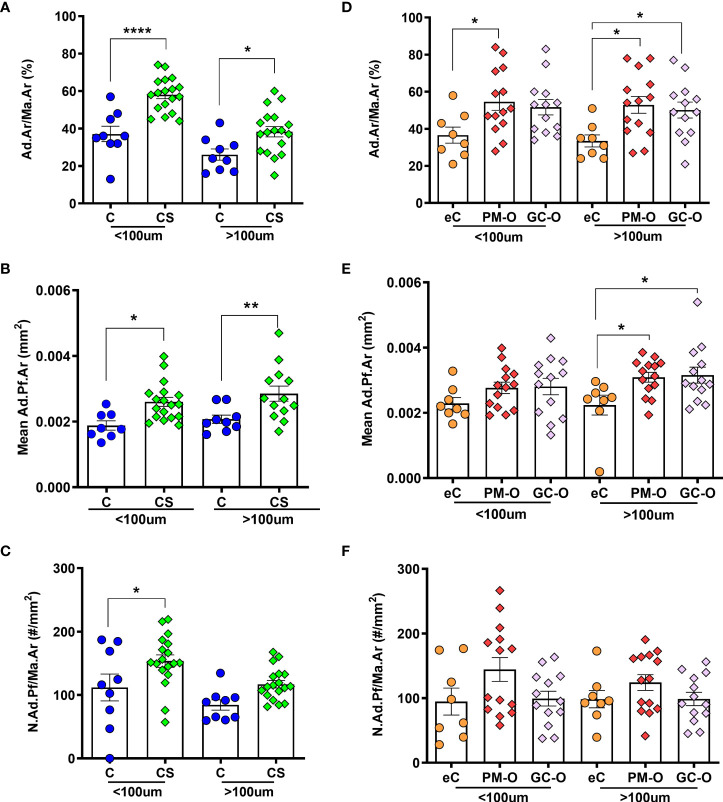
Effect of glucocorticoids on the spatial distribution of BMAT. **(A)** Ad.Ar/Ma.Ar is increased in CS compared with C, both within and above 100 µm from bone surface. **(B)** Mean Ad.Pf.Ar is also increased in CS irrespective of the spatial distribution. **(C)** Adipocyte density proximal to trabecular bone surface is increased in CS. **(D)** Ad.Ar/Ma.Ar is increased in patients with PM-O irrespective of the spatial distribution, but only above 100 µm from bone surfaces in GC-O biopsies. **(E)** Adipocyte profile size is increased distally to bone surfaces in both PM-O and GC-O. **(F)** Adipocyte profile density remains unchanged in patients with PM-O and GC-O, irrespective of the spatial distribution. Data are presented as individual biopsies and mean ± SEM. Data are analyzed by repeated measures two-way ANOVA followed by Bonferroni correction for multiple comparisons: *p < 0.05; **p < 0.01; ****p < 0.0001.

Next, the relative BMAT content in patients with increased GC levels was evaluated according to its proximity to trabecular bone surfaces. For this, Ad.Ar/Ma.Ar and mean Ad.Pf.Ar and N.Ad.Pf/Ma.Ar were characterized within and above 100 µm from trabecular and endocortical bone surfaces. The spatial analyses demonstrated that biopsies from patients with CS present a significant increase in Ad.Ar/Ma.Ar (**Figure 5A**) [p=0.0001 by two-way ANOVA; Bonferroni *post-hoc*: C vs CS <100 µm p<0.0001; >100 µm p=0.0140] and mean Ad.Pf.Ar (**Figure 5B**) [p=0.0045 by mixed-effects model; Bonferroni *post-hoc*: C vs CS <100 µm p=0.0195; >100 µm p=0.0043], independent of their proximity to bone surfaces. In contrast, the spatial density of adipocytes was heterogenous and significantly increased in endosteal bone surfaces (**Figure 5C**), but not above 100 µm from the bone surface [p=0.0092 by two-way ANOVA; Bonferroni *post-hoc*: C vs CS <100 µm p=0.0257; >100 µm p=0.096]. PM-O patients also presented heterogeneity in the spatial distribution of adipocytes in relation to bone surfaces. Patients with GC-O only displayed increased Ad.Ar/Ma.Ar and N.Ad.Pf/Ma.Ar above 100 µm from bone surface (**Figures 5D, E**) [p=0.021; eC vs GC-O <100 µm p=0.064, eC vs PM-O <100 µm p=0.020; eC vs GC-O >100 µm p=0.036, eC vs PM-O >100 µm p=0.011 by two-way ANOVA followed by Bonferroni´s multiple comparison] and Ad.Pf.Ar [p=0.030; eC vs GC-O <100 µm p=0.26, eC vs PM-O <100 µm p=0.31; eC vs GC-O >100 µm p=0.016, eC vs PM-O >100 µm p=0.0025 by two-way ANOVA followed by Bonferroni´s multiple comparison], while N.Ad.Pf/Ma.Ar remained unchanged (**Figure 5F**) [p=0.093]. PM-O patients displayed increased Ad.Ar/M a.Ar irrespective of their proximity to bone (**Figure 5D**), but mean Ad.Pf.Ar was only increased above 100 µm from the bone surface (**Figure 5E**). Overall, these results demonstrate alterations in the spatial distribution of BMAT in patients with hypercortisolemia, which is different than that of GC-treated patients.

To further characterize the spatial heterogeneity in BMAT content in the different patient cohorts, we evaluated the correlation of each analyzed parameter (i.e. Ad.Ar/Ma.Ar, mean Ad.Pf.Ar and N.Ad.Pf/Ma.Ar) proximal and distal to the bone surface, including the MixC group as a combination of all healthy individual biopsies analyzed (C and eC).

**Figure 6:**
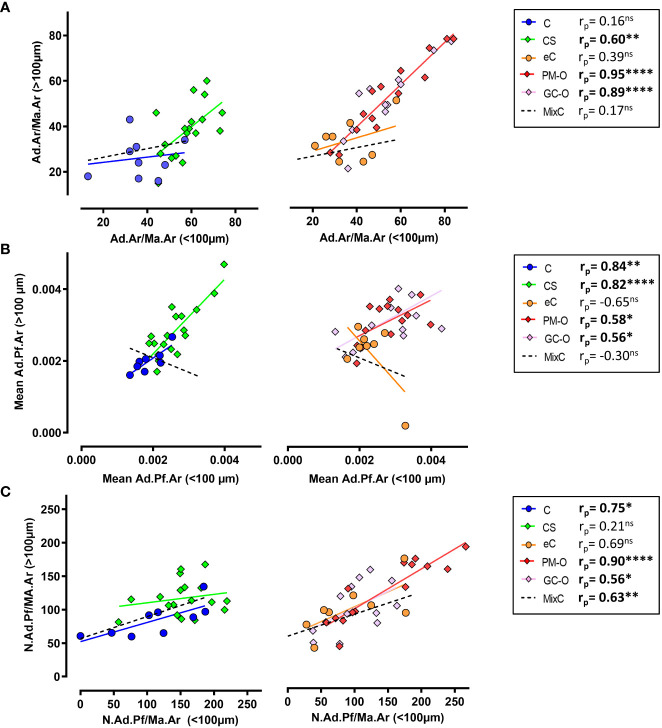
Spatial correlation of BMAT content characterization. **(A)** Correlation analysis between Ad.Ar/Ma.Ar proximal (<100 µm) or distal (>100 µm) to trabecular bone surfaces. **(B)** Correlation analysis between the spatial distribution of mean Ad.Pf.Ar relative to bone surface. **(C)** Correlation analysis of N.Ad.Pf/Ma.Ar distribution in relation to bone surface. Symbols represent individual patients and solid lines indicate simple linear regression analyses. Data are analyzed by Pearson (r_r_) correlation. *p < 0.05; **p < 0.01, ****p < 0.0001. ns, non significant.

The Ad.Ar/Ma.Ar within and above 100 µm of trabecular bone surfaces was significantly correlated in biopsies from patients with elevated glucocorticoids (i.e. CS and GC-O) and PM-O, but not in any of the control groups (**Figure 6A**), suggesting a disease-induced effect of spatial adipocyte accumulation. Analyses of mean Ad.Pf.Ar showed a significant correlation within and above 100 µm of bone surface in all groups but the healthy eC and MixC (**Figure 6B**), while N.Ad.Pf/Ma.Ar within and above 100 µm from bone was significantly correlated in osteoporotic patients and healthy C and MixC patient cohorts (**Figure 6C**).

Overall, our results indicate that, in physiological situations, the adipose content is primarily situated proximal to trabecular bone surfaces, while the mean Ad.Pf.Ar and N.Ad.Pf/Ma.Ar varies with age; however, the spatial distribution of BMAT is skewed in high GC environments.

### 3.4 Abundance of BMAT correlate with canopy coverage above bone remodeling events

**Figure 7:**
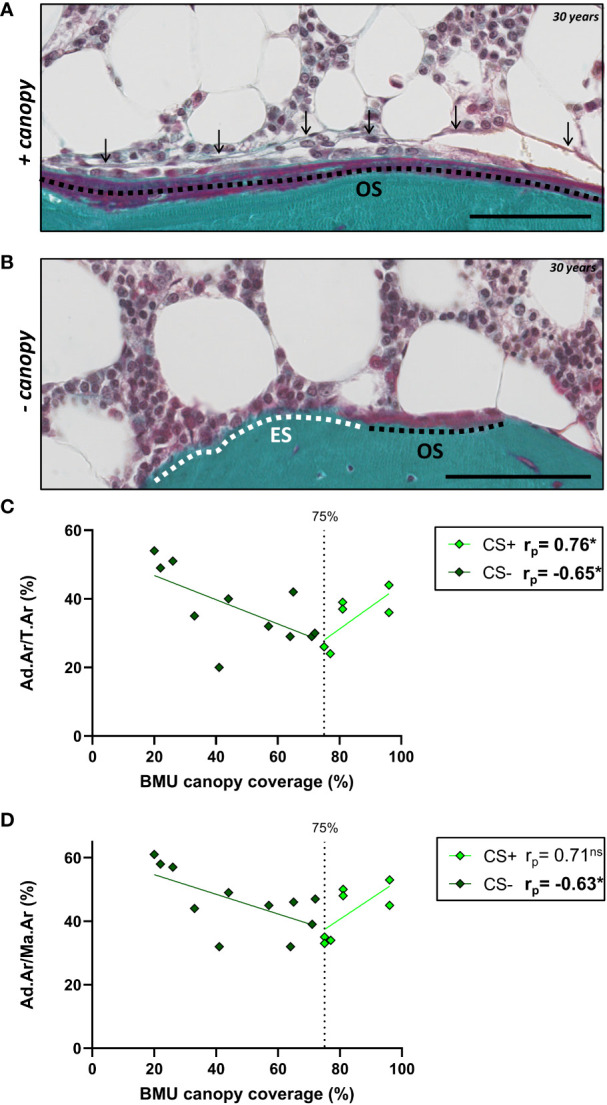
BMU canopy coverage and its correlation with BMAT parameters. **(A, B)** Images from Masson-trichrome stained section of a 30 years old CS patient showing osteoid surface (OS) covered by a canopy (arrows) **(A)**, and eroded surfaces (ES) and OS without any canopy coverage **(B)**. Scale bars are 100µm. **(C, D)** Graph showing that the Ad.Ar/T.Ar and Ad.Ar/Ma.Ar have a V-shaped correlation with the BMU canopy coverage. Data are analyzed by Pearson (r_p_) correlation: *p < 0.05. ns, non significant. This and additional correlation are listed in **Table 2**.

Next, we hypothesized that the described GC-induced alterations of BMAT correlate with changes in the trabecular bone remodeling and canopy coverage. To test this, we compared our CS BMAT data with the percentage of trabecular bone surface covered by osteoid surfaces (OS/BS) and eroded surface (ES/BS), and the canopy coverage above these bone surfaces [OS, ES and BMU (ES+OS)], illustrated in **Figure 7A**. In CS, the Ad.Ar/T.Ar and Ad.Ar/Ma.Ar had a V-shaped correlation with the BMU canopy coverage when dividing the CS patient into CS+ (i.e. ≥75% canopy coverage) and CS- (i.e. <75% canopy coverage) as previous (20) (**Figures 7B–D**), which was not the case for the mean Ad.Pf.Ar and N.Ad.Pf/Ma.Ar (**Table 2**). In contrast, the BMU and OS canopy coverage had a borderline significant (p=0.058) and significant (p=0.034) correlation with mean Ad.Pf.Ar when including all CS patients (**Table 2**). None of the BMAT parameters correlated with the OS/BS and ES/BS. In controls, none of the BMAT parameters showed any correlation with any of the latter bone and canopy parameters (**Table 2**).

**Table 2:** Impact of BMAT fraction on bone remodeling in patients with CS.

			Ad.Ar/T.Ar			Ad.Ar/Ma.Ar		Mean Ad.Pf.Ar			N.Ad.Pf/Ma.Ar		
			R_p_	P-value		R_p_	P-value	R_p_		P-value	R_p_		P-value
**C**	**OS/BS**		0.31	0.49		0.38	0.40	0.08		0.86	0.27		0.56
	**ES/BS**		-0.36	0.42		-0.41	0.36	-0.32		0.42	-0.32		0.49
	**BMU canopy coverage**		-0.49	0.26		-0.16	0.73	0.38		0.40	-0.66		0.11
	**OS canopy coverage**		-0.29	0.53		-0.06	0.90	0.32		0.49	-0.46		0.30
	**ES canopy coverage**		-0.02	0.97		0.11	0.81	0.45		0.31	-0.16		0.73
**CS**	**OS/BS**		-0.20	0.42		-0.30	0.22	-0.35		0.16	-0.19		0.45
	**ES/BS**		0.07	0.78		0.01	0.99	-0.08		0.76	0.02		0.94
	**BMU canopy coverage**		-0.43	0.08		-0.39	0.11	-0.47		0.06	-0.02		0.94
	**OS canopy coverage**		-0.44	0.07		-0.38	0.13	**-0.52**		**0.03**	0.01		0.97
	**ES canopy coverage**		0.00	1.00		-0.11	0.68	0.11		0.67	-0.28		0.27
	**CS (+)**	**BMU canopy coverage**	**0.76**	**0.05**		0.71	0.07	0.09		0.86	0.26		0.58
		**OS canopy coverage**	**0.79**	**0.01**		0.52	0.13	0.17		0.65	0.30		0.44
		**ES canopy coverage**	0.24	0.47		0.27	0.42	0.30		0.37	0.20		0.56
	**CS (–)**	**BMU canopy coverage**	**-0.65**	**0.03**		**-0.63**	**0.04**	-0.41		0.24	-0.48		0.13
		**OS canopy coverage**	-0.52	0.15		-0.66	0.08	-0.50		0.21	-0.45		0.22
		**ES canopy coverage**	-0.48	0.27		-0.52	0.23	0.02		0.98	-0.45		0.31

Table showing the correlation between BMAT parameters and histomorphometric bone and canopy parameters in CS patients and controls, as previously described (20). Data are analyzed by Pearson (r_p_) correlation. Red indicates borderline significant and bold indicate significant correlations.

Having previously established a significant correlation between serum cortisol levels and histological adiposity parameters in patients with CS (**Figure 3**), our next step was to evaluate whether canopy coverage also correlates with systemic cortisol levels. Our results demonstrate a significant association between the serum cortisol levels and canopy coverage in CS+ patients, but not in CS- patients (**Table 3**).

**Table 3:** Relationship between serum cortisol levels and canopy coverage in patients with CS.

	S-cortisol						
	**CS (+)**	Pearson r	P-value	**CS** (–)	Pearson r	P-value	
**BMU canopy coverage**		0.5940	0.1596		**-0.8506**	**0.0009**	
**OS canopy coverage**		**0.6672**	**0.0496**		**-0.7850**	**0.0122**	
**ES canopy coverage**		0.2455	0.4668		**-0.7994**	**0.0310**	

Table showing the correlation between canopy coverage parameters and serum cortisol levels in patients with CS. CS(+) indicates > 75% canopy coverage and CS (–) indicates < 75% canopy coverage. Data are analyzed by Pearson (r_p_) correlation. Red and bold indicates indicate significant correlations.

## 4 Discussion

CS is a disorder characterized by increased endogenous cortisol production that commonly induces the development of an osteoporotic-like condition (20, 24, 32, 33). As glucocorticoids are known to promote differentiation of SSPCs towards the adipogenic lineage (38, 39), alterations in BMAT in the bones of patients with CS are common (20, 25, 30–32). However, little is known about the differential effect of increased endogenous vs. exogenous glucocorticoids on BMAT content, and its effect of bone remodeling. In this histomorphometric study, we evaluate trephine iliac crest bone biopsies from patients with CS and matched controls from the BMAT perspective and compare the results to those of elderly patients with GC-O and their matched healthy and PM-O controls.

Our results suggest that both endogenous and exogenous glucocorticoids may induce BMAT accumulation, which is mediated solely by hypertrophy in GC-O, but both by hypertrophy and hyperplasia in CS. While increased BMAT in response to elevated glucocorticoid levels is well established (21, 25, 30, 33, 38, 39), animal models have suggested a temporal regulation of adipocyte hypertrophy and hyperplasia (40). This is however difficult to study in the human setting due to the limited access to histological samples. Instead, we examined the relationship between glucocorticoid levels and BMAT accumulation and demonstrated a significant association between serum cortisol, Ad.Ar/Ma.Ar and Ad.Pf.Ar in patients with CS. However, GC-O patients showed that BMAT parameters are independent of the accumulated glucocorticoid dose, suggesting irreversible bone damage following hormonal therapy. This is a crucial problem, as undertreatment of GC-O is widely documented (41, 42) and it has been reported that less than 20% of GC-treated patients are prescribed bone protective medication along with the steroid prescription (43, 44).

In this study, we evaluated cohorts that represent two differential age ranges: middle age (C and CS) and elderly (eC, GC-O and PM-O) patients. Our analyses demonstrate that age is significantly correlated with Ad.Ar/Ma.Ar and Ad.Pf.Ar in healthy individuals, as expected (45, 46), but this correlation is altered in an environment with increased glucocorticoid exposure. To further understand the hormonal effect of BMAT, we characterized the adipose content from a spatial perspective in relation to trabecular bone surfaces. Overall, our results indicate pronounced BMAT accumulation proximal to bone surfaces (<100 µm) in patients with CS, which was mediated by both adipocyte hypertrophy and hyperplasia. In the marrow >100 µm from bone surface, only Ad.Pf.Ar was affected. These results suggest the existence of a spatial gradient of steroid-increased adiposity. One possible explanation is the reported higher density of endosteal SSPCs compared to central marrow (47) and the fact that these endosteal SSPCs have adipogenic potential (48). Additionally, this higher density of endosteal SSPCs could relate to the presence of the bone marrow envelope at the endosteum. In contrast, the CG-O cohort showed increased BMAT content, which was mediated solely by adipocyte hypertrophy. These results were further corroborated by the spatial analyses of marrow >100 µm of trabecular bone surface, while proximal to bone marrow, BMAT remained unchanged. It is important to point out that the 100 µm threshold for proximity used in this study was randomly selected, as the results could be affected by different proximity thresholds. In this study, we selected a 100 µm radius consistently with our previous studies of spatial vascularization (49) and innervation of the human bone (50). Moreover, it could be speculated that differences in BMAT between patients with CS and GC-O are due to higher apoptosis response to exogenous GC than endogenous cortisol; however, further studies are necessary to investigate this possibility.

To better understand the effect of altered BMAT on bone metabolism in patients with CS, we examined the correlation between BMAT parameters and our previous histomorphometric characterization of bone formation, resorption and canopy coverage in these samples (19, 20). Only when dividing the CS patients according to their canopy coverage (CS(+) versus CS (–)) (20), we observed a correlation between canopy coverage and Ad.Ar/T.Ar and Ad.Ar/M.Ar, as the correlation was V-shaped. The inverse V-shaped correlation of CS+ and CS- patients emphasize that these two subgroups of patients have a very different link between the BMAT and canopy coverage, as previously shown to be the case the bone formation parameters (20). This is in line with our proposed model, in which canopy cells are a source of osteoblastic progenitors that are rejuvenated by SSPC (18). Additionally, that osteopenic and osteoporotic conditions is associated with a loss of these canopy cells and a delayed transition from erosion to formation (this study) (16, 19–21). The negative correlation between adiposity and canopy coverage in CS- patients may reflect the SSPC preferentially differentiate towards the adipogenic lineage instead of rejuvenating the canopy cells, subsequently impairing bone formation due to lack of osteoprogenitors in these patients. This line of research would support investigating SSPC as a druggable target to prevent bone disease in patients with CS. On the other hand, the positive correlation between adiposity and canopy coverage in CS+ patients may reflect that these patients have a sufficient capacity of SSPC to both rejuvenate canopy cells and bone marrow adipocytes. The CS+ and CS- patient have similar serum glucocorticoid levels, but the CS- patients have been reported to be more sensitive to serum glucocorticoid levels from a canopy perspective (19). The histomorphometric studies are however limited to the transiliac bone biopsies, and may not fully reflect a systemic effect of cortisol increase on bone metabolism in patients with CS, especially the vertebrae where most fractures occur. Another important limitation of this study is the complex comparison of endogenous circulating cortisol in CS patients and exogenously administered GC in postmenopausal women, with different dosing and treatment periods.

As the field of histomorphometry continues to advance, automated systems that evaluate all cells and surfaces present in a bone biopsy are becoming more widely available (51, 52) and may uncover correlations between BMAT and canopy coverage that are missed in this study. Another limitation of this study is that it is performed on tissue sections where we conduct 2D histomorphometry; as such, the investigated 2D adipocyte profiles underestimate the true size of the 3D adipocytes in all groups, but the differences in 2D adipocyte profile size reflect a 3D size difference. Moreover, the estimated 2D densities of adipocyte profiles are size-weighted, meaning that this parameter is partly influence by the adipocyte size, as bigger adipocytes have a larger chance of being included in the analysis, limiting the information can be inferred to a 3D context.

In conclusion, this study provides a comprehensive characterization of BMAT accumulation in patients with CS, PM-O and GC-O that demonstrates increased adiposity, hypertrophy and hyperplasia. Moreover, we show that adiposity and hypertrophy in CS patients are directly correlated with serum cortisol and that hyperplasia occurs proximal to trabecular bone surface, and a V-shaped correlation with changes in the canopy coverage. The disparity in the pattern of adiposity in the marrow of patients with increase endogenous or exogenous cortisol highlights the differential mechanisms of glucocorticoid-induced adiposity, which may pose differential therapeutic targets to improve bone disease in these patient populations.

## Data availability statement

The data analyzed in this study is subject to the following licenses/restrictions: Human data; all data is available upon reasonable request. Requests to access these datasets should be directed to marta@forens.au.dk.

## Ethics statement

The studies involving humans were approved by Danish National Committee on Biomedical Research Ethics. The studies were conducted in accordance with the local legislation and institutional requirements. The ethics committee/institutional review board waived the requirement of written informed consent for participation from the participants or the participants’ legal guardians/next of kin because samples were collected from Danish pathological biobanks.

## Author contributions

Project conceptualization and funding acquisition: TLA, JMD, MK, AJ. Data obtention and analyses: NS, CA, PJ, EH, JB, MDC. Manuscript writing: NS, MDC. All authors contributed to manuscript revision and editing and approved the submitted version.
