# Supplementary material for: Disturbed bone marrow adiposity in patients with Cushing’s syndrome and glucocorticoid- and postmenopausal- induced osteoporosis
Source: Front Endocrinol (Lausanne). 2023 Oct 10;14:1232574. doi: 10.3389/fendo.2023.1232574 (PMC10597666; doi:10.3389/fendo.2023.1232574)
Supplement: Supplementary file 1 [file Table_1.docx]

|  |  | CS | | GC-O | | PM-O | |
| --- | --- | --- | --- | --- | --- | --- | --- |
|  |  | **Slope** | **Intercept** | **Slope** | **Intercept** | **Slope** | **Intercept** |
| C | **Ad.Ar/T.Ar** | 0.3032 | 0.0023 | - | - | - | - |
|  | **Ad.Ar/Ma.Ar** | 0.0931 | 0.0003 | - | - | - | - |
|  | **Mean Ad.Pf.Ar** | 0.8615 | 0.0031 | - | - | - | - |
|  | **N.Ad.Pf/Ma.Ar** | 0.4129 | 0.0059 |  |  |  |  |
| eC | **Ad.Ar/T.Ar** | - | - | 0.1966 | 0.0169 | 0.8858 | 0.0072 |
|  | **Ad.Ar/Ma.Ar** | - | - | 0.6923 | 0.0221 | 0.7625 | 0.0149 |
|  | **Mean Ad.Pf.Ar** | - | - | 0.0493 |  | 0.6850 | 0.0087 |
|  | **N.Ad.Pf/Ma.Ar** | - | - | 0.8282 | 0.8098 | 0.7291 | 0.2587 |
| MixC | **Ad.Ar/T.Ar** | 0.1416 | 0.0008 | 0.2167 | 0.0032 | 0.6928 | 0.0011 |
|  | **Ad.Ar/Ma.Ar** | 0.0513 | <0.0001 | 0.6128 | 0.0052 | 0.6018 | 0.0027 |
|  | **Mean Ad.Pf.Ar** | 0.8802 | 0.0003 | 0.0105 |  | 0.8394 | 0.0025 |
|  | **N.Ad.Pf/Ma.Ar** | 0.2822 | 0.0055 | 0.7523 | 0.8176 | 0.3067 | 0.2058 |

**Supplementary table 1.** Summary of slope and intercept *p*-values for the correlation of BMAT parameters and age. Red indicates significance.

|  |  | CS | | GC-O | | PM-O | |
| --- | --- | --- | --- | --- | --- | --- | --- |
|  |  | **Slope** | **Intercept** | **Slope** | **Intercept** | **Slope** | **Intercept** |
| C | **Ad.Ar/Ma.Ar** | 0.0950 | 0.6878 | - | - | - | - |
|  | **Mean Ad.Pf.Ar** | 0.3833 | 0.5531 | - | - | - | - |
|  | **N.Ad.Pf/Ma.Ar** | 0.3990 | 0.0285 |  |  |  |  |
| eC | **Ad.Ar/Ma.Ar** | - | - | 0.0442 |  | 0.0176 |  |
|  | **Mean Ad.Pf.Ar** | - | - | 0.0134 |  | 0.0037 |  |
|  | **N.Ad.Pf/Ma.Ar** | - | - | 0.8710 | 0.8952 | 0.4217 | 0.8837 |
| MixC | **Ad.Ar/Ma.Ar** | 0.0859 | 0.9898 | 0.0064 |  | 0.0018 |  |
|  | **Mean Ad.Pf.Ar** | 0.0005 |  | 0.0442 |  | 0.0317 |  |
|  | **N.Ad.Pf/Ma.Ar** | 0.2702 | 0.1817 | 0.4788 | 0.3666 | 0.0584 | 0.1354 |

**Supplementary table 2.** Summary of slope and intercept *p*-values for the correlation of the BMAT spatial distribution in relation to trabecular bone surface. Red indicates significance.

|  | | Ad.Ar/T.Ar | | Mean Ad.Pf.Ar | | N.Ad.Pf/Ma.Ar | |
| --- | --- | --- | --- | --- | --- | --- | --- |
|  |  | Pearson r | P-value | Pearson r | P-value | Pearson r | P-value |
| C | **OS/BS** | 0.1406 | 0.6315 | 0.2653 | 0.3594 | 0.3226 | 0.2607 |
|  | **ES/BS** | 0.0329 | 0.9109 | 0.1695 | 0.5623 | -0.1586 | 0.5881 |
| GC-O | **OS/BS** | -0.2621 | 0.387 | -0.2036 | 0.5047 | -0.077 | 0.8024 |
|  | **ES/BS** | -0.5712 | 0.0415* | -0.1235 | 0.6878 | -0.0864 | 0.7788 |

**Supplementary table 3. Impact of BMAT fraction on bone remodeling in patients with GC-O**. Correlation table between BMAT characterization and histomorphometric bone parameters in GC-O patients, as previously described (1). AV/TV= Adipose tissue volume per total volume. OS/BS=fraction of osteoid surface per bone surface. ES/BS= fraction of eroded surface per bone surface. Red indicates statistical significance (p<0.05).
